# Draft genome sequence of *Denitratisoma* sp. strain agr-D3, isolated from common reed using a droplet-based cultivation method

**DOI:** 10.1128/mra.01012-25

**Published:** 2026-02-09

**Authors:** Tomoki Iwashita, Manabu Kanno, Yuri Ota, Satoko Matsukura, Masamune Morita, Akira Sasaki, Naohiro Noda, Tomoki Nishioka, Yuga Hirakata, Hideyuki Tamaki

**Affiliations:** 1 Biomanufacturing Process Research Center, National Institute of Advanced and Industrial Science and Technology (AIST), Tsukuba, Ibaraki, Japan; 2 Molecular Biosystems Research Institute, National Institute of Advanced and Industrial Science and Technology (AIST), Tsukuba, Ibaraki, Japan; Indiana University, Bloomington, Indiana, USA

**Keywords:** *Denitratisoma*, *Phragmites australis*, droplet-based cultivation

## Abstract

We report the draft genome sequence of *Denitratisoma* sp. strain agr-D3, isolated from common reed using a droplet-based cultivation method. The draft genome is 3,082,957 bp with a G + C content of 63.6%. The genome was predicted to contain 2,914 coding sequences and 4 rRNA genes.

## ANNOUNCEMENT

Members of the genus *Denitratisoma* are anaerobic bacteria known to perform denitrification and steroid degradation (1, 2). Recent culture-independent studies suggest that these bacteria are primarily found in the rhizosphere (3–5), but their interactions with host plants remain poorly understood. Plant-associated bacteria can exert negative, neutral, or positive effects on plant growth, and characterizing their traits provides insights into ecology, environmental engineering, and agriculture. Here, we describe the draft genome sequence of a novel isolate, *Denitratisoma* sp. strain agr-D3, from the rhizosphere of the common reed.

Root samples of *Phragmites australis* were collected from Lake Kasumigaura, Tsuchiura City, Ibaraki, Japan (36.06° N, 140.22° E), and homogenized as previously described (6). The homogenates were filtered through a 0.22 μm membrane filter (Merck, Germany), and microorganisms trapped on the filter were suspended in 5 mL of 1/100-strength tryptic soy broth (Becton, Dickinson and Company, USA). The suspension was adjusted to 1.1 × 10^6^ cells/mL, and water-in-oil droplets were generated using a DG8 cartridge (Bio-Rad, USA) and an On-chip Droplet Generator (On-chip Biotechnologies), as described previously (7). Droplets were stored in 2 mL tubes and incubated at 25°C for 35 days. After incubation, droplets were dispensed into 96-well microplates containing 180 µL of 1/100-strength tryptic soy broth supplemented with 1.5% agar using the On-chip SPiS (On-chip Biotechnologies), based on a previous study (8). The microplates were incubated at 25°C for 28 days, and a single colony, designated strain agr-D3, was isolated. For genome sequencing, strain agr-D3 was cultivated in R2A medium (9) at 25°C with shaking at 150 rpm for 5 days. Cells were collected by centrifugation, and genomic DNA was extracted using the Extrap Soil DNA Kit Plus v.2 (BDL, Japan), according to the manufacturer’s protocol. Whole-genome shotgun sequencing was performed using the DNBSEQ-T7 platform (MGI Tech). A short-read library was prepared from fragmented DNA using the MGIEasy Fast FS DNA Library Prep Set v.2.0 (MGI Tech) and circularized with the MGIEasy Dual Barcode Circularization Kit (MGI Tech). Paired-end sequencing (2 × 150 bp) was performed using the DNBSEQ-T7 platform with a High-throughput Pair-End Sequencing Primer Kit (MGI Tech). A total of 2,473,610 raw reads were quality filtered using fastp v.0.23.2 (10) and assembled using SPAdes v.3.13.0 (11). Genome annotation was performed using the DDBJ Fast Annotation and Submission Tool Pipeline v.1.3.4 (https://dfast.ddbj.nig.ac.jp/) (12). Genome completeness and contamination were assessed using CheckM v.1.2.2 (13). All tools were run with default parameters unless otherwise specified. The 16S rRNA gene sequence derived from the agr-D3 genome shared the highest similarity (96.10%) with *Denitratisoma oestradiolicum* AcBE2-1^T^ (accession number AY879297) in the EzBioCloud (14). The draft genome of *Denitratisoma* sp. strain agr-D3 was assembled into six contigs totaling 3,082,957 bp, with a G + C content of 63.6% and an N50 of 735,769 bp and was predicted to contain 2,914 coding sequences, 4 rRNA genes, and 49 tRNA genes from a total of 2,318,700 reads.

## Data Availability

The draft genome sequence and annotations of *Denitratisoma* sp. strain agr-D3 have been deposited in DDBJ/EMBL/GenBank under accession numbers BAAGIA010000001, BAAGIA010000002, BAAGIA010000003, BAAGIA010000004, BAAGIA010000005, and BAAGIA010000006. The genome sequence has also been submitted to the SRA under BioSample accession number SAMD00859346 and BioProject accession number PRJDB19912. Raw sequence data for strain agr-D3 were deposited under DRA accession number DRR633685.
